# Effect of a Prior Two-Hour Postural Test on Seated Saline Suppression Test Results in Patients Predominantly Evaluated for Adrenal Incidentalomas

**DOI:** 10.3390/jcm15124410

**Published:** 2026-06-07

**Authors:** Krzysztof C. Lewandowski, Katarzyna Wojciechowska-Durczyńska, Kinga Krawczyk-Rusiecka, Marta Mikulak, Ewa Bieniek, Wojciech Horzelski

**Affiliations:** 1 Faculty of Medicine—Collegium Medicum, Department of Internal Medicine, Endocrinology & Diabetes, Mazovian University in Plock, Dąbrowskiego 2, 09-402 Plock, Poland; m.mikulak@mazowiecka.edu.pl (M.M.);; 2 Department of Paediatric and Adult Endocrinology, Medical University of Lodz, 90-419 Lodz, Poland; 3 Department of Endocrinology & Metabolic Diseases, “Polish Mother’s” Memorial Hospital Research Institute, Rzgowska 281/289, 93-338 Lodz, Poland; 4 Faculty of Mathematics and Computer Science, University of Lodz, Stefana Banacha 22, 90-238 Lodz, Poland

**Keywords:** aldosterone, renin, primary aldosteronism, hyperaldosteronism, saline suppression test, postural test

## Abstract

**Background/Objectives**: The Seated Saline Suppression Test (SSST) is widely used for investigation of primary hyperaldosteronism (PA); however, the impact of a one-hour sitting period prior to SSST (as recommended by the Endocrine Society, with a 7.8 ng/dL aldosterone cut-off) on the test outcome remains unclear. **Methods**: In 82 individuals (23 males) aged 57.3 ± 12.6 years, BMI 27.1 ± 5.1 kg/m^2^, where the majority were diagnosed with adrenal incidentalomas (n = 75), we performed an SSST preceded by a 120 min postural test (first protocol), or immediately after waking (second protocol, a cross-over design). **Results**: Though aldosterone and renin concentrations before the onset of SSST were higher after the first protocol (*p* < 0.001), an overall suppression of aldosterone was not different after the first versus the second protocol (6.34 ± 0.68 ng/dL (mean ± SEM) versus 5.74 ± 0.66 ng/dL, *p* = 0.124). There was no significant difference between the number of individuals with aldosterone > 10 ng/dL, i.e., nine (11%) for the first protocol versus eight (9.75%) for the second protocol, *p* = 1.00, and for the “grey zone” aldosterone of 7.8–10 ng/mL after SSST (five (6.1%) in each group, *p* = 1.0). Aldosterone-to-direct-renin ratio (ADR ratio) was higher in those who failed to suppress aldosterone (*p* < 0.001) but offered a poor prediction of SSST outcome with the lowest ADR ratio of 0.398 ng/dL/µIU/mL in those with aldosterone > 7.8 ng/dL, and the highest ADR ratio of 26.04 ng/dL/µIU/mL for those with aldosterone < 7.8 ng/dL on SSST. **Conclusions**: Two-hour postural test before SSST does not significantly impact aldosterone suppression after saline infusion, while the ADR ratio does not fully discriminate between those who suppress and those who do not suppress aldosterone secretion on SSST, at least in those with adrenal incidentalomas.

## 1. Introduction

Primary hyperaldosteronism remains both the most common cause of hormone-dependent hypertension and one of the most underdiagnosed diseases in clinical medicine. Vaidaya A & Carey RM state in their review [1] that only about 1% of cases of primary aldosteronism are correctly diagnosed. Some authors [2] also indicate that “unnecessary complexity in definition and diagnosis constitutes a barrier to wider clinical care”. For instance, even though aldosterone-to-renin ratio (ARR) has been proposed as a screening test [3], the test is rarely performed outside Endocrine Departments, and, at least in our country, is virtually never performed in departments of cardiology or internal medicine in the absence of frank hypokalemia. There is no definite agreement as to the precise cut-off point for ARR, while Kline GA states in his paper that “*once measured, interpretation of the ARR can be a formidable task. Plasma aldosterone levels may be reported either in ng/dL or pmol/L. Renin may be reported according to plasma renin activity (PRA) in ng/L/s, ng/mL/h or pmol/L/min, or alternately as a direct renin concentration in mU/L or ng/dL. Depending on the assays in use, the case-defining ARR can be numerically highly variable from 1.6 to 1000*”. This is precisely the reason why physicians outside the field of endocrinology are either reluctant to measure ARR (as they cannot interpret it) or misinterpret the results after looking them up in a medical textbook, while these cut-off points were often defined in some university departments many years prior to publication of the above-mentioned textbook and were based on different assays than those used nowadays.

As a result of this conundrum, patients in Poland, and particularly those with adrenal adenomas (incidentalomas), are generally referred directly to Endocrine Departments for further investigations, usually without any screening endocrine tests. Furthermore, access to renin assays is very limited outside teaching hospitals. On the strength of the above, it is imperative to reach a definite diagnosis during a single hospital stay. Hence, in practice, we generally perform dynamic testing in most cases of adrenal adenomas or suspected hormone-related hypertension. In this setting, the recent Endocrine Society guidelines [3] advocate that, if a Seated Saline Suppression Test (SSST) is performed, then “after sitting for 1 h, blood should be drawn to mark t = 0”. This indicates that an obligatory period of either ambulation or at least in a seated position is recommended prior to the SSST.

On the strength of the above, we have therefore endeavoured to assess whether the period of an upright posture, and in our case a formal two-hour postural test, can indeed influence the results of the subsequent SSST.

## 2. Materials and Methods

This retrospective study included 82 subjects (23 males), aged 57.3 ± 12.6 (mean ± SD), BMI 27.1 ± 5.1 kg/m^2^. Of these, 75 (91.4%) patients had a history of adrenal adenomas (bilateral in 11 cases), while the remaining seven patients had a history of poorly controlled hypertension. All subjects had a history of hypertension, though of various severity, and creatinine concentrations within the reference range. Patients with adrenal adenomas were referred from either GP clinics or other departments where adrenal lesions (incidentalomas) were detected on imaging studies performed for non-endocrine indications. These usually included some form of abdominal discomfort or routine follow-up of patients with non-specific pulmonary abnormalities, usually due to past COVID-19 infection. None of these patients had adrenal vein sampling prior to our investigations. Adrenal surgery was subsequently conducted in three cases of unilateral adenomas, where the presence of aldosterone-producing adenomas was confirmed according to the current histopathology consensus [4]. Patients with resistant hypertension (n = 7) were referred from endocrine clinics, however, without prior renin testing. All patients had treatment modification for at least two weeks prior to testing that included a change from an antihypertensive medication to an alpha-blocker (doxazosin) ± a calcium channel blocker (slow-release verapamil). In three cases of patients receiving either spironolactone, this medication was stopped about four weeks prior to hospitalisation, and potassium supplements were administered. This was arranged by means of a formal written request to the family physician that was handed to the patient on the day that the date for the admission was established. Hypokalemia was corrected in all (four) cases.

In all subjects, a two-hour postural test was performed (1st protocol) followed by two litres of normal saline (0.9%) infusion over 4 h (500 mL/h for 4 h), while maintaining a seated position, as recommended by the Endocrine Society [3]. Hence, blood samples for the measurements of aldosterone and renin were taken at time 0 min (supine), 120 min (after a period of ambulation or a sitting position) and finally after four hours of saline infusion. Following a one-day wash-out period (usually dedicated to 24 h urinary collection for methoxy-catecholamines), the second saline infusion was performed (2nd protocol), where a blood sample was drawn for aldosterone and renin at time 0 (supine position), i.e., after an overnight sleep (usually 7–8 h) and subsequently after four hours of saline infusion. This implied that there was no prior period of ambulation. Each patient was subsequently asked to assume a sitting position and a saline infusion was started.

As described before [5,6], aldosterone concentrations were measured by an automated chemiluminescence immunoassay Liason^®^ DiaSorin Inc. (Saluggia, Italy), in which, according to the manufacturer leaflet, the intraassay variation is reported as 3.5% at 6.8 ng/dL and 1.8% at 28.8 ng/dL. Total coefficients of variation were 9.5% at 6.8 ng/dL and 5.6% at 28.8 ng/dL. Manufacturer references were as follows: aldosterone supine 1.17–23.6 ng/dL and aldosterone upright 2.21–35.3 ng/dL. Renin was also measured by means of Liason^®^ DiaSorin Inc. (Italy) immunoassay, with the intraassay variation of 12.4% at 13.2 µIU/mL and 4.7% at 260.3 µIU/mL. Total coefficients of variation were 0.6% at 13.2 µIU/mL and 1.7% at 260.3 µIU/mL, where manufacturer reference ranges for renin were reported as renin supine 2.8–39.9 µIU/mL for supine, and 4.4–46.1 µIU/mL for an upright position, respectively.

Significant hypokalemia (defined as serum potassium < 3.5 mmol/L) was either excluded or corrected by potassium supplementation prior to both tests. The study was limited to individuals whose initial renin concentrations were either within or below the reference range, thereby excluding cases of secondary hyperaldosteronism.

### 2.1. Interpretation of Results Obtained

According to recent Endocrine Society Guidelines [3], primary aldosteronism might be excluded when aldosterone concentration after saline infusion is below 217 pmol/L (7.8 ng/dL). Notably, however, we have also taken into account older recommendations [7], where primary hyperaldosteronism was excluded for aldosterone < 5 ng/dL (140 pmol/L) and confirmed for aldosterone concentrations > 10 ng/dL (280 pmol/L). Hence, we divided the patients into those in whom the diagnosis of primary hyperaldosteronism was very likely (plasma aldosterone > 10 ng/dL (280 pmol/L), described as “possible hyperaldosteronism”); those in the “grey zone”, described as “possibly some autonomy in aldosterone secretion” (plasma aldosterone ≥ 7.8 ng/dL (217 pmol/L) but ≤10 ng/dL (280 pmol/L)); and those in whom primary hyperaldosteronism has been excluded, i.e., for plasma aldosterone < 7.8 ng/dL (217 pmol/L).

In addition, we have compared the number of individuals in the “lower grey zone”, i.e., those with aldosterone concentration between 5 and 7.8 ng/dL (140–217 pmol/L).

### 2.2. Ethical Approval

On admission, all patients provided written permission that their data might be presented anonymously for research and training purposes in accordance with Regulation EU 2016/679 of the European Parliament and of the Council of 27 April 2016 on the protection of natural persons with regard to processing personal data and on the free movement of such data.

The study was also submitted for formal ethical approval by The Ethics Committee of the Polish Mother’s Memorial Hospital Research Institute, and it was concluded that formal approval was deemed unnecessary (Decision nr KB-43/2024), as the study was designed as optimisation of standard clinical practice; therefore, it did not fulfil the criteria of “medical experiment” according to article 21, clause 1, of the Bill on The Profession of Medical and Dental Practitioners from 5 December 1996, latest version in The Journal of Enactments of the Polish Parliament (Dz. U. z 2022 r. position 617).

### 2.3. Statistical Analysis

Statistical analysis was performed using MedCalc^®^ Statistical Software version 20.1.10 (MedCalc Software Ltd., Ostend, Belgium). Continuous variables were tested for normality of distribution using the Shapiro–Wilk test. As most variables did not follow a normal distribution, results are presented as mean ± standard error of the mean (SEM), median, standard deviation (SD), and 95% confidence intervals (95% CI), as appropriate.

Comparisons between paired continuous variables (e.g., hormone concentrations before and after postural test, and after 1st versus 2nd saline infusion) were performed using the Mann–Whitney U test, as detailed in Table 1B, and the Wilcoxon signed-rank test. Comparisons between categorical variables (number of patients above or below predefined aldosterone cut-off points) were performed using the Chi-square (χ^2^) and McNemar’s tests. A two-tailed *p*-value < 0.05 was considered statistically significant.

## 3. Results

**Figure 1:**
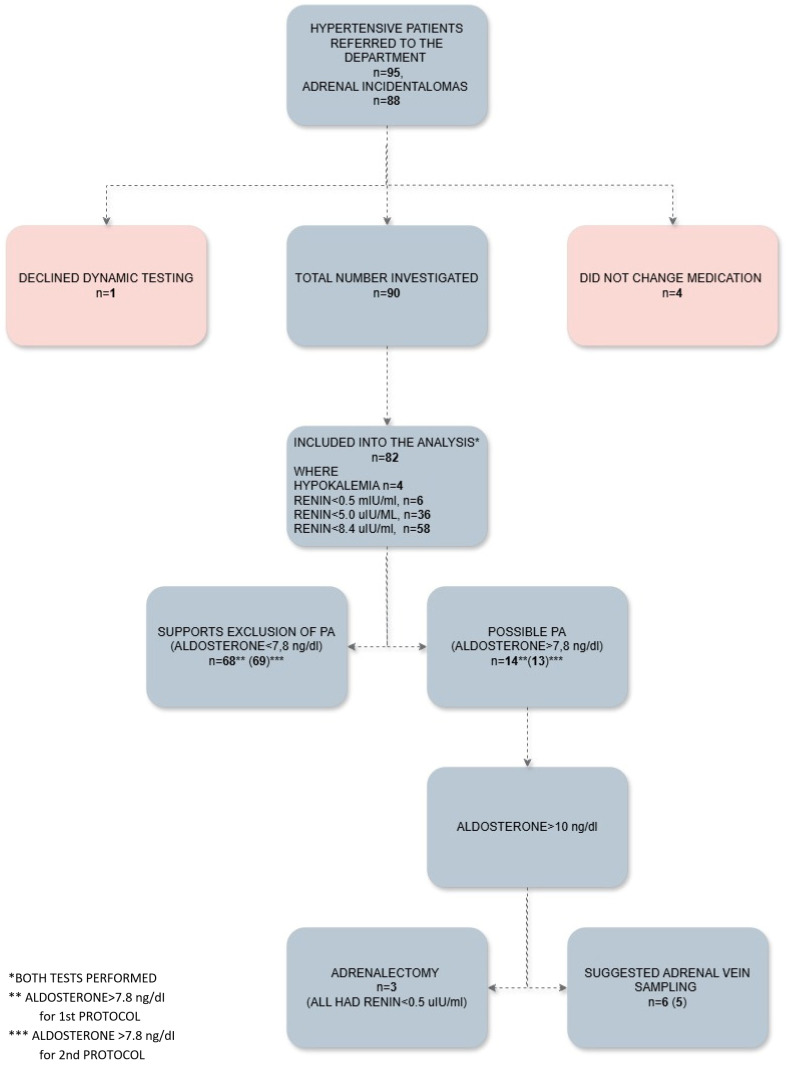
Flow diagram of the recruitment process and clinical outcomes of patients investigated during the study. The term “adrenalectomy” applies to removal of solitary (i.e., unilateral) adrenal adenomas.

A flow diagram of recruitment and investigation process is presented in Figure 1.

**Figure 2:**
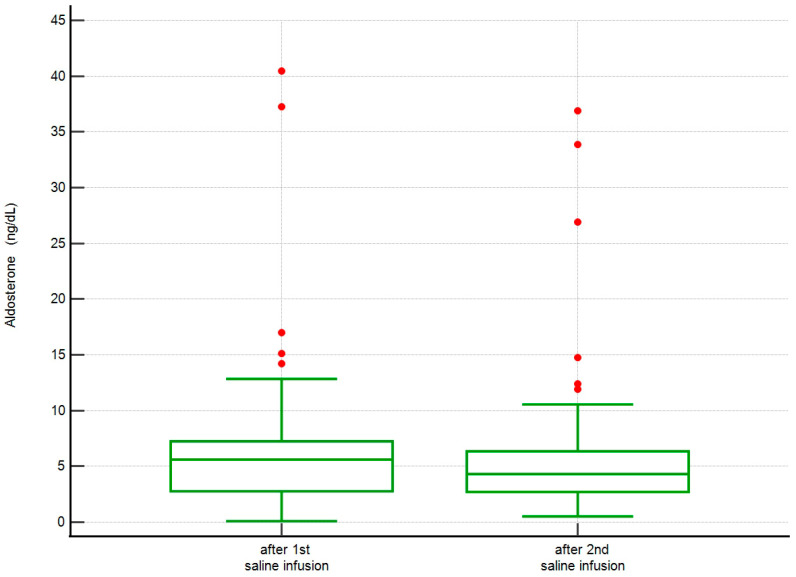
Comparison of aldosterone concentrations [ng/dL] after SSST preceded by a two-hour postural test (after the 1st saline infusion) and not preceded by any period in an upright posture (after the 2nd saline infusion). Box depicts the 1st–3rd interquartile range. Red dots represent outliers.

Initially, six patients had suppressed renin (<0.5 µIU/mL), while low renin concentrations were observed either in 58 patients (for 8.4 µIU/mL renin cut-off) or in 36 patients for 5 µIU/mL renin cut-off. There were only three patients with renin concentrations above 20 µg/dL (i.e., within the upper half of the reference range), all of whom showed subsequent aldosterone suppression below 7.8 ng/dL after both tests. Notably, initial renin concentrations were not known till the results of both tests were available, as all samples of an individual patient were analyzed once a week in a single batch. Low potassium (corrected by potassium supplements) was observed in four cases. Results of the study are presented in Table 1A,B and Figure 2. There was a significant increase in aldosterone and renin concentrations following a 120 min period of an upright posture (i.e., standing or sitting position), *p* < 0.0001; however, an overall suppression of aldosterone secretion was either not different after the first protocol versus the second protocol (6.34 ± 0.68 ng/dL (mean ± SEM) versus 5.74 ± 0.66 ng/dL, *p* = 0.124 (Mann–Whitney U test)) or showed a trend towards slightly lower values (*p* = 0.08 for Wilcoxon test (mean ± SEM)). There was also a trend towards lower renin concentrations after the second protocol (7.38 ± 0.81 µIU/mL versus 5.24 ± 0.46 µIU/mL, *p* = 0.064 (Mann–Whitney U test)), which reached significance (*p* < 0.001) when the Wilcoxon signed-rank test was applied.

Supine aldosterone concentrations before the 1st saline infusion (first protocol) were not different from aldosterone concentrations before the 2nd saline infusion (second protocol), i.e., 8.92 ± 1.07 ng/dL, versus 9.50 ± 1.20 ng/dL, *p* = 0.96 (Wilcoxon signed-rank test), compared to renin 8.02 ± 0.77 µIU/mL versus 8.32 ± 0.93 µIU/mL, *p* = 0.38. The same applied to the aldosterone-to-direct-renin (ADR) ratio (i.e., 4.1704 ± 1.52 ng/dL/µIU/mL versus 5.005 ± 1.92 ng/dL/µIU/mL, *p* = 0.99, with virtually identical medians (1.11 ng/dL/µIU/mL and 1.05 ng/dL/µIU/mL) for 0’ supine versus before 2nd saline infusion, respectively—Table 1B). In our opinion, this implicated an adequate wash-out period between the 1st and 2nd tests. Comparison of aldosterone concentrations after the 1st and 2nd saline infusion is also presented in Figure 2.

Having defined a non-inferiority margin of 0.74 ng/dL, calculated from a 9.5% aldosterone assay coefficient of variation (7.8 ng/dLx 0.095 = 0.74 ng/dL), the mean difference in aldosterone concentration between the first and the second infusion was found to be 0.604 ng/dL (95% CI: 0.058 to 1.149). The lower bound of the confidence interval was above the predefined non-inferiority margin (−0.74 ng/dL), indicating that the second infusion was not inferior to the first.

Regarding the clinical outcome of patients with non-suppressed aldosterone after SSST, three patients had subsequent surgery that confirmed aldosterone-producing adenomas. Adrenal vein sampling was recommended for all individuals with aldosterone > 10 ng/dL who also received treatment with either spironolactone or eplerenone.

Analysis of the obtained data according to predefined cut-off points is presented in Table 2. There was no difference in the number of individuals with plasma aldosterone concentrations above 10 ng/dL (280 pmol/L) regardless of the period of ambulation prior to the SSST (nine versus eight, *p* = 0.82), in the “grey zone” (aldosterone 7.8–10 ng/dL, 217–280 pmol/L, five in each group, *p* = 1.0), or the combined group of those with aldosterone concentrations above 7.8 ng/dL (217 pmol/L), i.e., 14 versus 13, *p* = 1.0. Differences between the two protocols pertained only to the number of patients in the former “lower grey zone” and those with aldosterone < 5.0 ng/dL.

**Figure 3:**
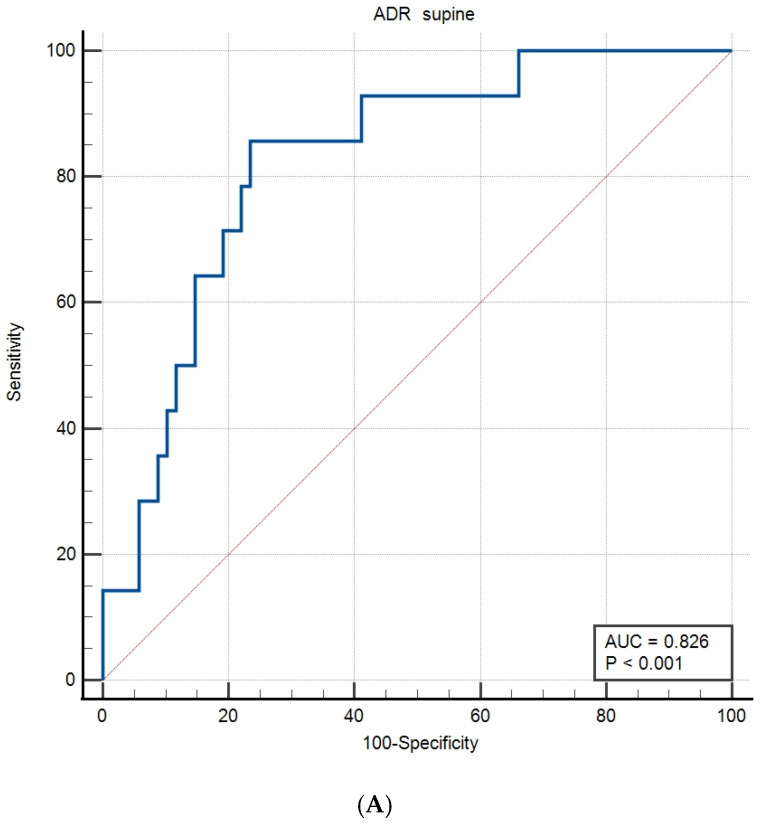

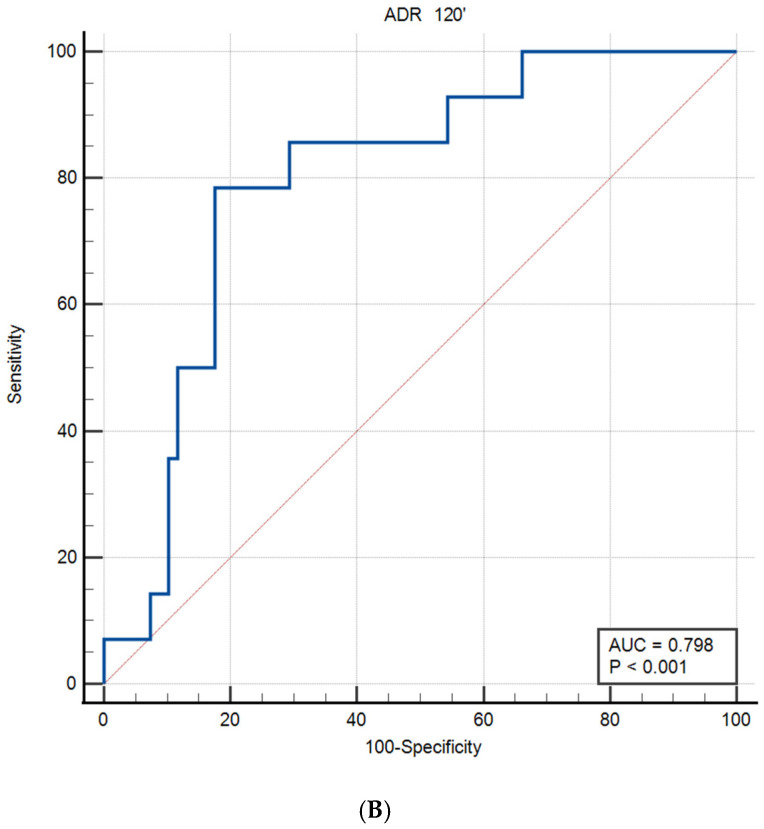
(**A**) ROC analysis for ADR ratio [ng/dL/µIU/mL] in supine position (before the 1st saline infusion) for prediction of failure of suppression of aldosterone concentrations below 7.8 ng/dL during SSST. Blue line represents a ROC curve, where the true positive rate (Sensitivity) is plotted in function of the false positive rate (100-Specificity) for different cut-off points. Each point on the ROC curve represents a sensitivity/specificity pair corresponding to a particular decision threshold. Red line represents a diagonal generated by statistical programme. (**B**) ROC analysis for ADR ratio [ng/dL/µIU/mL] in an upright position (after 120 min of the postural test and before the 1st saline infusion) for prediction of failure of suppression of aldosterone concentrations below 7.8 ng/dL during SSST.

Aldosterone-to-direct-renin ratio (ADR ratio) was significantly higher for both supine and upright positions (i.e., after the postural test) in those who failed to suppress aldosterone concentrations below 7.8 ng/dL (217 pmol/L) during SSST, i.e., 13.75 ± 8.42 ng/dL/µIU/mL versus 2.12 ± 0.45 ng/dL/µIU/mL, *p* < 0.0001, for supine and 5.96 ± 3.73 ng/dL/µIU/mL versus 1.78 ± 0.51 ng/dL/µIU/mL, *p* = 0.0005. Nevertheless, there was a significant overlap between the two groups, as there were subjects with relatively high ADR ratios who suppressed aldosterone concentrations below 7.8 ng/dL (217 pmol/L), i.e., the highest values of 20.32 ng/dL/µIU/mL and 26.04 ng/dL/µIU/mL, for supine and upright positions, respectively. In fact, only two out of 14 subjects who failed to suppress aldosterone concentrations below 7.8 ng/dL had ADR ratios above these cut-offs. On the other hand, there were subjects with rather low ADR ratios who failed to suppress aldosterone concentration on SSST below 7.8 ng/dL, i.e., who had lowest values of 0.631 ng/dL/µIU/mL and 0.398 ng/dL/µIU/mL, for supine and upright positions, respectively (Table 3). On the strength of these findings, we have performed a formal ROC analysis of the predictive value of ADR ratios for failure of suppression of aldosterone concentrations below 7.8 ng/dL during the SSST. Results of this analysis are presented in Figure 3A,B and the corresponding Table 4A,B.

## 4. Discussion

Dynamic tests for primary hyperaldosteronism have undergone significant evolution. For instance, the postural test was originally conducted for four hours [7,8], with the two-hour test only employed after 1978. The Saline Suppression Test was generally performed in a recumbent position till 2018 [9], i.e., till the study of Stowasser M et al. [10] was published.

Our data are actually fully consistent with the results of the study by Stowasser M et al. [10], who compared performance in the seated (SSST) versus recumbent (RSST) Saline Suppression Test in 100 subjects (n = 82 in our study). ROC analysis in the above-mentioned study predicted an optimal cut-off for aldosterone concentrations of 162 pmol/L for the SSST and 106 pmol/L for the RSST. Notably, both of these values are lower than the current cut-off point of 217 pmol/L (7.8 ng/dL) suggested by the Endocrine Society. Hence, with an application of this higher cut-off point, it could have been predicted that the period of ambulation prior to SSST is probably unnecessary, while our study merely confirms this presumption.

We note that the period of ambulation, and in our case a formal two-hour postural test, prior to the SSST might potentially increase the number of subjects in the “lower grey zone” (5.0–7.8 ng/dL), but this does not influence the final interpretation of the test results, with application of the current cut-off point suggested by the Endocrine Society (217 pmol/L (7.8 ng/dL)), as the “lower grey zone” has actually been abolished. Notably, in our study, there were more subjects (39 versus 23) in the post-infusion aldosterone concentration range of 5.0–7.8 ng/dL, when the SSST was preceded by the postural test (*p* = 0.01). Furthermore, the number of individuals investigated in our study (n = 82) was similar to the number of subjects (n = 80) analysed by Thuzar M. et al. [11], i.e., in the study from which the current Endocrine Society cut-off point of 217 pmol/L (7.8 ng/dL) for aldosterone suppression on SSSP was derived, with a quoted specificity of 86.7% and 86.2% sensitivity.

There is also the question of whether a one-day wash-out period was long enough to ensure an adequate discriminatory value of the second SSST, i.e., without any period of prior ambulation. Stowasser M et al. [10] applied at least a two-week wash-out between the seated and the recumbent SSST. Such an approach was feasible in a research setting, but not in a standard clinical practice, as this would require two separate hospital admissions. Nevertheless, an inadequate wash-out (i.e., with persistent salt overload after the first SSST) would have presumably led to a smaller number of individuals with aldosterone concentrations > 7.8 ng/dL after the second SSST. Yet, only a single individual with aldosterone concentration just above 10 ng/dL moved to a 7.8–10 ng/dL group (10.38 ng/dL versus 9.95 ng/dL), and such a difference falls within the assay coefficient of variation. The same applies to a single individual who had an aldosterone concentration of 7.85 ng/dL after the first SSST and 7.36 ng/dL after a second SSST. Hence, the lack of any significant difference in the above-mentioned number of individuals with aldosterone > 7.8 ng/dL after the second SSST, despite such a short wash-out period, actually confirms the validity of our findings. This is further supported by the lack of difference in supine aldosterone concentrations as well as ADR ratio before the two tests. Another issue pertains to the initial renin concentrations in our study cohort. Thuzar M. et al. [11] performed SSST only in individuals with renin concentration below 8.4 µIU/mL. Given the negligible availability of renin assays in the outpatient setting, such an approach would be, however, impractical in our case, when the cost of a screening hospital admission plus the second hospital admission for the SSST would be much higher than the full investigation during a single hospital stay. Notably, 70.7% of our patients had initial renin concentrations below 8.4 µIU/mL, while the elimination of three individuals with renin concentrations in the upper half of the reference range (all of whom showed aldosterone suppression <7.8 ng/dL on both SSSTs) did not influence the results of our study. We need to stress, however, that our patient cohort was severely slanted towards those with adrenal adenomas, so findings might be different, e.g., in an unselected population of young patients with hypertension.

Despite these limitations, while taking into account the complexity of diagnosis of primary aldosteronism with a plethora of various units and cut-off points that is daunting for many endocrinologists, let alone physicians outside the field of endocrinology, we do think that any simplification of current diagnostic protocols should increase the number of patients who are investigated for primary aldosteronism. Abolishing the mandatory ambulatory period prior to the SSST would simplify diagnostic work-up, both in the hospital and in the outpatient settings, such as Endocrine Day Rooms, where patients can be assessed and can start the test immediately after arrival, i.e., without any period of walking or sitting in the clinic corridor.

Though not the subject of our study, we have chosen to perform a formal two-hour postural test prior to saline infusion rather than ensuring just a one-hour ambulation period. Hence, we can conclude that if a two-hour period of upright posture did not influence SSST outcomes, then it is difficult to surmise that a shorter (i.e., one hour) period would have yielded different results. Furthermore, the role of the postural test has evolved over the years. Initially, the test was supposed to identify patients with a flat aldosterone response to ambulation (or even a decline in aldosterone concentrations), which was presumed to be consistent with classical aldosterone-producing adenomas (Conn’s syndrome), where aldosterone concentrations were more dependent on ACTH than on renin as the main stimulant [7,8]. Recently, there has been some renewed interest in this test [12,13]. A postural test with a flat aldosterone response has been reported to be useful, particularly in cases of unilateral adrenal adenomas [14]. The main challenge of the postural test is, however, poor definition as to what actually should be considered as a normal response. Hence, a “normal” posture-dependent increase in aldosterone concentrations can range from above 30% [15] to above 50%, with direct renin concentrations < 10 ng/L, or ≤1 ng/mL/hour for plasma renin activity, and a cortisol increase from ≤50% [16], up to 74% (with reported specificity of 59%) [17] or even as high as up to 200% [18]. Notably, in the latter study [18], the authors identified five out of 19 subjects with posture-responsive, surgically confirmed adrenal adenoma, but if a 30% aldosterone increase cut-off had been applied, then posture-dependent adrenal adenomas would have been present in 14 out of 19 patients [73.6%]. In our study, 22 out of 82 patients had an increase in aldosterone concentrations below 30%, and 24 out of 84 had an increase < 50%. This exceeded the number of subjects with either confirmed (eight or nine patients, depending on the protocol) or suspected primary hyperaldosteronism (five in each group). As a consequence, we have abandoned the idea of separate analysis of postural test data, considering that “flat” aldosterone response in our opinion does not obviate the need for a subsequent dynamic test.

Our study had several limitations, including the lack of initial screening with an aldosterone/direct renin ratio (ADR ratio) for the selection of patients for subsequent SSST as a confirmatory study. This is important in view of recommendations that all young hypertensive individuals, as well as those with resistant hypertension, should be screened for primary hyperaldosteronism, while widespread application of dynamic testing is not feasible from a population perspective. Such an approach has been, however, determined by very poor availability of renin measurements in outpatient settings, where renin assays were available only in two endocrine clinics in the Lodz district (with a catchment area of about two million) and in only one clinic in the Plock district (with a catchment area of about 0.5 million). In our opinion, this is not necessarily a result of inadequate funding but rather a reflection of poor understanding of the actual prevalence of primary hyperaldosteronism and the value of performing screening tests among physicians outside the endocrine field.

In such settings, as depicted in the flow diagram, most patients with adrenal adenomas or suspicion of hormone-dependent hypertension are referred for hospital admission without any screening tests (or with aldosterone measurement alone). Furthermore, even in teaching hospital settings, results of aldosterone and renin measurements are not readily available, as the whole batch of samples is usually analysed every 7–10 days. Hence, in our opinion, it was better to adjust antihypertensive medication and to perform a full diagnostic work-up during a single hospital stay in order to provide a comprehensive answer to the referring physician, rather than to perform just screening tests, and then to arrange the second hospital admission for selected patients. Notably, our study cohort consisted of over 90% of individuals with adrenal adenomas, so more research is needed to determine whether our results can be extrapolated to a wider population. Nevertheless, as discussed below, performance of the ADR ratio appears to be a rather poor screening tool, at least in individuals with adrenal incidentalomas (Figure 3A,B).

Furthermore, we have data based on our own measurements in healthy subjects, with an upper cut-off point of an aldosterone/direct renin ratio of 1.5359 [ng/dL/µIU/mL] in healthy non-pregnant women [5]. This is less than the recently reported age-dependent ratios of 2.3, 2.8, 3.1, and 3.6 ng/dL/µIU/mL for those ≤39, 40–49, 50–59, and ≥60 years, respectively [19]. We have therefore retrospectively analysed the ADR ratio in our patients. The ADR ratio was indeed significantly higher in those who failed to suppress aldosterone below 7.8 ng/dL (*p* < 0.001, see Table 3). Nevertheless, if we take into account the data of patients both in a supine position and after 120 min of ambulation, then there are patients with a “normal” ADR ratio who fail to suppress aldosterone secretion after saline infusion (with the lowest value for ADR ratio of 0.398 ng/dL/µIU/mL). According to the current understanding, a failure of adequate suppression of aldosterone secretion in the setting of salt loading denotes that aldosterone secretion is at least partially autonomous from renin and therefore angiotensin release [20]. Furthermore, ROC analysis provided rather poor results for sensitivity of the above-quoted ADR ratios (i.e., 2.3, 2.8, 3.1, and 3.6 ng/dL/µIU/mL) [19]. For instance, even for the highest of these cut-off points (3.6 ng/dL/µIU/mL), we had only about 42% sensitivity for prediction of failure of aldosterone suppression in a supine position (Figure 3A and Table 4A). This fell to a very poor sensitivity (about 7%) for the ADR ratio after 120 min of the postural test. Taking into account that a trend for diagnosis of primary aldosteronism [3] moves away from the widespread use of dynamic testing, it is imperative to standardise both recommended cut-off points and assay units for calculation of ADR ratio in order to avoid confusion among the clinicians. The same applies to the conversion factor from plasma renin activity to direct renin concentrations that is quoted as 12 in some papers [21], or “8.2” by other authors [22].

On the other hand, some patients with a high ADR ratio (the highest value of 26.04 ng/dL/µIU/mL) demonstrated good suppression of aldosterone secretion after SSST. Hence, in our opinion, a universal application of the aldosterone-to-renin ratio as a screening test should probably be used more as a guide rather than as a rigid cut-off point that would preclude patients from further investigations in terms of performing dynamic tests. Such an approach should at least be applied to those with confirmed adrenal adenomas. This is because adrenal adenomas were present in 75 out of 82 (91.5%) of our patients of our study cohort. Notably, a similar approach to ours was also reported in other centres [23], where all patients with adrenocortical incidentaloma or bilateral adrenocortical hyperplasia (49.2%), hypokalemic hypertension (32.2%), and resistant hypertension (18.6%) underwent confirmatory testing for primary hyperaldosteronism regardless of their aldosterone-to-renin ratio.

## 5. Conclusions

In summary, our study demonstrated that the two-hour postural test prior to SSST does not significantly affect the performance of the SSST in terms of the number of individuals who fail to suppress aldosterone concentrations < 7.8 ng/dL, i.e., the cut-off suggested by the Endocrine Society [3]. Hence, an obligatory period of ambulation prior to the SSST can probably be safely abolished at least in those with adrenal incidentalomas. As the ADR ratio does not accurately predict who would fail to suppress aldosterone secretion on SSST, the application of this tool as a screening test requires more detailed research, particularly in patients with confirmed adrenal adenomas.

## Figures and Tables

**Table 1:** (**A**) Aldosterone [ng/dL] and renin concentrations [µIU/mL] before and after postural test (0’ supine and 120’ upright), after the 1st saline infusion of two litres over four hours (1st protocol) and before the 2nd saline infusion, and after the 2nd saline infusion of two litres over 4 h (2nd protocol without prior ambulation). (**B**) Statistical significance for the alterations of aldosterone [ng/dL] and renin concentrations [µIU/mL] presented in (**A**) before and after the postural test (0’ supine and 120’ upright), after the 1st saline infusion of two litres over four hours (1st protocol) and before the 2nd saline infusion, and after the 2nd saline infusion of two litres over 4 h (2nd protocol without prior ambulation), as well as for the aldosterone-to-direct-renin (ADR) ratio, calculated by means of the Wilcoxon signed-rank test. Statistical significance between variables compared at various time-points can be seen at the crossing of the horizontal lines for the given time-point for each variable that is compared with values for the same variable at a different time-point as denoted at the top of the table.

(**A**)					
**ALDOSTERONE [ng/dL]**	**0’ Supine**	**120’ Upright**	**After 1st** **Saline Infusion**	**Before 2nd** **Saline Infusion**	**After 2nd** **Saline Infusion**
**Sample size**	82	82	82	82	82
**Lowest value**	1.370	2.120	0.10	1.50	0.50
**Highest value**	67.83	73.50	40.46	76.20	36.92
**Arithmetic mean**	8.92	16.27	6.34	9.50	5.74
**95% CI for the Arithmetic mean**	6.79 to 11.05	13.72 to 18.83	4.99 to 7.69	7.11 to 11.89	4.42 to 7.05
**Median**	6.97	13.53	5.61	6.40	4.31
**95% CI for the median**	6.02 to 7.93	12.08 to 15.37	4.312 to 63.08	6.05 to 8.54	3.52 to 5.05
**Interquartile range**	5.65	10.7	4.45	5.74	3.52
**Variance**	942.48	1348.26	375.99	1187.87	359.21
SD *	9.71	11.61	6.13	10.89	5.99
SEM **	1.07	1.28	0.68	1.20	0.66
**RENIN [µIU/mL]**	**0’ Supine**	**120’ Upright**	**After 1st** **Saline Infusion**	**Before 2nd** **Saline Infusion**	**After 2nd** **Saline Infusion**
**Lowest value**	0.500	0.500	0.50	0.50	0.50
**Highest value**	33.24	116.10	50.48	37.40	19.96
**Arithmetic mean**	8.02	23.86	7.38	8.32	5.24
**95% CI for the Arithmetic mean**	6.48 to 9.55	19.03 to 28.71	5.78 to 8.99	6.46 to 10.18	4.32 to 6.17
**Median**	5.800	16.290	5.810	5.530	4.160
**95% CI for the median**	4.76 to 8.11	12.39 to 20.10	4.28 to 8.13	4.07 to 7.02	3.14 to 5.49
**Interquartile range**	7.49	29.5	7.89	7.97	5.25
**Variance**	48.66	485.34	53.39	70.67	17.77
SD *	6.976	22.031	7.307	8.407	4.216
SEM *	0.770	2.433	0.807	0.934	0.465
(**B**)					
**ALDOSTERONE**		**120 min upright**	**after 1st saline infusion**	**before 2nd saline infusion**	**after 2nd saline infusion**
**supine**		**<0.0001**	**0.0001**	**0.962**	**<0.0001**
**120 min upright**			**<0.0001**	**<0.0001**	**<0.0001**
**after 1st saline infusion**				**<0.0001**	**0.079**
**before 2nd saline infusion**					**<0.0001**
**RENIN**		**120 min upright**	**after 1st saline infusion**	**before 2nd saline infusion**	**after 2nd saline infusion**
**supine**		**<0.0001**	**0.1092**	**0.388**	**<0.0001**
**120 min upright**			**<0.0001**	**<0.0001**	**<0.0001**
**after 1st saline infusion**				**0.835**	**<0.0001**
**before 2nd saline infusion**					**<0.0001**
**ALDOSTERONE-to-DIRECT-RENIN RATIO (ADR ratio) [ng/dL/** **µ** **IU/mL]**		**120 min upright**	**after 1st saline infusion**	**before 2nd saline infusion**	**after 2nd saline infusion**
**supine**		**0.0002**	**0.0008**	**0.9981**	**0.9579**
**120 min upright**			**0.3080**	**0.0002**	**0.0126**
**after 1st saline infusion**				**0.0025**	**0.0021**
**before 2nd saline infusion**					**0.7360**

* Standard Deviation, ** Standard Error of the Mean.

**Table 2:** Number of individuals with and without adequate suppression of aldosterone concentrations after SSST (two litres of 0.9% saline over four hours) with a prior 120 min postural test (1st protocol) and without ambulation (2nd protocol).

	After 1st 0.9% Saline Infusion 1st Protocol	After 2nd 0.9% Saline Infusion 2nd Protocol	*p* (McNemar Test)
**all**	82	82	82
**Above 10 ng/dL** **(supports diagnosis of primary aldosteronism (PA))**	9	8	1.00
**7.8–10.0 ng/dL** **(“grey zone”—makes PA more likely)**	5	5	1.00
**Below 7.8 ng/dL** **(supports exclusion** **of PA)**	68	69	1.00
**5.0–7.8 ng/dL** **(former “lower grey zone”)**	39	23	0.01
**Less than 5.0 ng/dL**	34	51	0.01

**Table 3:** Aldosterone-to-direct-renin ratio (ADR ratio) [ng/dL/µIU/mL] before the 1st saline infusion in subjects who suppressed aldosterone secretion < 7.8 ng/dL (n = 68) and in those who failed to suppress it (n = 14), respectively, *p* < 0.0001, and *p* = 0.0005, for supine and upright positions, respectively. Numerical data were almost identical for ADR ratios before the second saline infusion (*p* = 0.9627, for comparison of ADR ratio in supine positions before the 1st and 2nd saline infusion, Wilcoxon signed-rank test).

ADR Ratio SUPINE POSITION	ABOVE 7.8 ng/dL	BELOW 7.8 ng/dL
Sample size	14	68
Lowest value	0.631	0.129
Highest value	121.14	20.32
Arithmetic mean	13.750	2.122
95% CI for the Arithmetic mean	−4.457 to 31.96	1.221 to 3.023
Median	3.36	0.879
95% CI for the median	2.11 to 8.76	0.66 to 1.26
SD	31.53	3.72
SEM	8.42	0.45
**ADR ratio UPRIGHT** **AFTER 120 min**	**ABOVE 7.8 ng/dL**	**BELOW 7.8 ng/dL**
Sample size	14	68
Lowest value	0.398	0.649
Highest value	54.343	26.040
Arithmetic mean	5.967	1.785
95% CI for the Arithmetic mean	−2.09 to 14.03	0.77 to 2.80
Median	2.467	0.568
95% CI for the median	1.950 to 3.215	0.405 to 0.974
SD	13.96	4.19
SEM	3.730	0.51

**Table 4:** (**A**) Sensitivity and specificity analysis of ADR ratio [ng/dL/µIU/mL] for the ROC curve presented in Figure 3A (supine position before the 1st saline infusion). (**B**) Sensitivity and specificity analysis of ADR ratio [ng/dL/µIU/mL] for the ROC curve presented in Figure 3B (in an upright position, i.e., after 120 min of the postural test and before the 1st saline infusion).

(**A—supine position**)						
**Criterion** **ADR Ratio** **[ng/dL/µIU/mL]**	**Sensitivity**	**95% CI**	**Specificity**	**95% CI**	**+LR**	**−LR**
≥0.128786251	100.00	76.8–100.0	0.00	0.0–5.3	1.00	
>0.516106804	100.00	76.8–100.0	33.82	22.8–46.3	1.51	0.00
>0.63115693	92.86	66.1–99.8	33.82	22.8–46.3	1.40	0.21
>1.069844358	92.86	66.1–99.8	58.82	46.2–70.6	2.26	0.12
>1.150080257	85.71	57.2–98.2	58.82	46.2–70.6	2.08	0.24
>1.895973154	85.71	57.2–98.2	76.47	64.6–85.9	3.64	0.19
>1.974789916	78.57	49.2–95.3	76.47	64.6–85.9	3.34	0.28
>2.120679887	78.57	49.2–95.3	77.94	66.2–87.1	3.56	0.27
>2.12371134	71.43	41.9–91.6	77.94	66.2–87.1	3.24	0.37
>2.166368515	71.43	41.9–91.6	80.88	69.5–89.4	3.74	0.35
>2.171764706	64.29	35.1–87.2	80.88	69.5–89.4	3.36	0.44
>2.665865385	64.29	35.1–87.2	85.29	74.6–92.7	4.37	0.42
>2.969565217	50.00	23.0–77.0	85.29	74.6–92.7	3.40	0.59
>3.296035242	50.00	23.0–77.0	88.24	78.1–94.8	4.25	0.57
>3.75862069	42.86	17.7–71.1	88.24	78.1–94.8	3.64	0.65
>3.855135135	42.86	17.7–71.1	89.71	79.9–95.8	4.16	0.64
>4.58	35.71	12.8–64.9	89.71	79.9–95.8	3.47	0.72
>5.18404908	35.71	12.8–64.9	91.18	81.8–96.7	4.05	0.71
>5.744047619	28.57	8.4–58.1	91.18	81.8–96.7	3.24	0.78
>6.9375	28.57	8.4–58.1	94.12	85.6–98.4	4.86	0.76
>9.634943182	14.29	1.8–42.8	94.12	85.6–98.4	2.43	0.91
>20.32	14.29	1.8–42.8	100.00	94.7–100.0		0.86
>121.14	0.00	0.0–23.2	100.00	94.7–100.0		1.00
(**B upright position**)						
**Criterion** **ADR**	**Sensitivity**	**95% CI**	**Specificity**	**95% CI**	**+LR**	**−LR**
≥0.064873418	100.00	76.8–100.0	0.00	0.0–5.3	1.00	
>0.377990431	100.00	76.8–100.0	33.82	22.8–46.3	1.51	0.00
>0.379826087	92.86	66.1–99.8	33.82	22.8–46.3	1.40	0.21
>0.459587021	92.86	66.1–99.8	45.59	33.5–58.1	1.71	0.16
>0.476092896	85.71	57.2–98.2	45.59	33.5–58.1	1.58	0.31
>1.165333333	85.71	57.2–98.2	70.59	58.3–81.0	2.91	0.20
>1.168711656	78.57	49.2–95.3	70.59	58.3–81.0	2.67	0.30
>1.867454068	78.57	49.2–95.3	82.35	71.2–90.5	4.45	0.26
>2.211009174	50.00	23.0–77.0	82.35	71.2–90.5	2.83	0.61
>2.574468085	50.00	23.0–77.0	88.24	78.1–94.8	4.25	0.57
>2.941328175	35.71	12.8–64.9	88.24	78.1–94.8	3.04	0.73
>3.076397516	35.71	12.8–64.9	89.71	79.9–95.8	3.47	0.72
>3.23458445	14.29	1.8–42.8	89.71	79.9–95.8	1.39	0.96
>3.362521891	14.29	1.8–42.8	92.65	83.7–97.6	1.94	0.93
>3.480392157	7.14	0.2–33.9	92.65	83.7–97.6	0.97	1.00
>26.04	7.14	0.2–33.9	100.00	94.7–100.0		0.93
>54.342592593	0.00	0.0–23.2	100.00	94.7–100.0		1.00

## Data Availability

Data can be available on request from the corresponding author (K.C.L.).

## Abbreviations

The following abbreviations are used in this manuscript:

SSST	Seated Saline Suppression Test
ADR ratio	Aldosterone-to-direct-renin ratio
